# ECDC’s latest publications

**DOI:** 10.2807/1560-7917.ES.2018.23.13.180329-2

**Published:** 2018-03-29

**Authors:** Eurosurveillance editorial team

**Affiliations:** 1 European Centre for Disease Prevention and Control (ECDC), Stockholm, Sweden

**Keywords:** infectious diseases, public health, Europe

**Rapid risk and outbreak assessments**

Rapid risk assessment: Outbreak of yellow fever in Brazil, Third update.

Date of publication: 16 March 2018

https://ecdc.europa.eu/en/publications-data/rapid-risk-assessment-outbreak-yellow-fever-brazil-third-update

Rapid risk assessment: Outbreak of yellow fever in Brazil

Date of publication: 18 January 2018

https://ecdc.europa.eu/en/publications-data/rapid-risk-assessment-outbreak-yellow-fever-brazil-second-update

Joint Rapid Outbreak Assessment: Multi-country outbreak of *Salmonella* Agona infections linked to infant formula

Date of publication: 17 January 2018

https://ecdc.europa.eu/en/publications-data/joint-rapid-outbreak-assessment-multi-country-outbreak-salmonella-agona

Risk assessment for seasonal influenza, EU/EEA, 2017–2018

Date of publication: 20 December 2017

https://ecdc.europa.eu/en/publications-data/risk-assessment-seasonal-influenza-eueea-2017-2018

**Technical reports**

Guidelines for presentation of surveillance data

Date of publication: 14 March 2018

https://ecdc.europa.eu/en/publications-data/guidelines-presentation-surveillance-data

External quality assessment scheme for *Bordetella pertussis* serology 2016

Date of publication: 06 February 2018

https://ecdc.europa.eu/en/publications-data/external-quality-assessment-scheme-bordetella-pertussis-serology-2016

EU/EEA capacity for the surveillance of hepatitis B and C using molecular methods 

Date of publication: 23 January 2018

https://ecdc.europa.eu/en/publications-data/eueea-capacity-surveillance-hepatitis-b-and-c-using-molecular-methods

**Surveillance reports**

Tuberculosis surveillance and monitoring in Europe, 2018

Date of publication: 19 March 2018

https://ecdc.europa.eu/en/publications-data/tuberculosis-surveillance-and-monitoring-europe-2018

Monthly measles and rubella monitoring report, March 2018

Date of publication: 9 March 2018

https://ecdc.europa.eu/en/publications-data/monthly-measles-and-rubella-monitoring-report-march-2018

The European Union summary report on antimicrobial resistance in zoonotic and indicator bacteria from humans, animals and food in 2016

Date of publication: 27 February 2018

https://ecdc.europa.eu/en/publications-data/european-union-summary-report-antimicrobial-resistance-zoonotic-and-indicator-4

Monthly measles and rubella monitoring report, February 2018

Date of publication: 9 February 2018

https://ecdc.europa.eu/en/publications-data/monthly-measles-and-rubella-monitoring-report-february-2018

Influenza virus characterisation, Summary Europe, January 2018

Date of publication: 17 January 2018

https://ecdc.europa.eu/en/publications-data/influenza-virus-characterisation-summary-europe-december-2017

Monthly measles and rubella monitoring report, January 2018

Date of publication: 12 January 2018

https://ecdc.europa.eu/en/publications-data/monthly-measles-and-rubella-monitoring-report-january-2018

ECDC/EFSA joint report: Avian influenza overview September – November 2017

Date of publication: 22 December 2017

https://ecdc.europa.eu/en/publications-data/ecdcefsa-joint-report-avian-influenza-overview-september-november-2017

**Corporate publication**

ECDC international relations policy 2020

Date of publication: 29 January 2018

https://ecdc.europa.eu/en/publications-data/ecdc-international-relations-policy-2020

**Scientific peer-reviewed publications**

Publications by ECDC staff in scientific journals

Updated monthly

https://ecdc.europa.eu/en/publications-data?f%5b0%5d=output_types%3A1247

**Annual Epidemiological Report series on communicable diseases in Europe**

Seasonal influenza - Annual Epidemiological Report for 2016-17 season

Date of publication: 15 March 2018

https://ecdc.europa.eu/en/publications-data/seasonal-influenza-annual-epidemiological-report-2016-17-season

Zoonotic influenza - Annual Epidemiological Report for 2016

Date of publication: 15 March 2018

https://ecdc.europa.eu/en/publications-data/zoonotic-influenza-annual-epidemiological-report-2016

Tularaemia - Annual Epidemiological Report for 2015

Date of publication: 30 January 2018

https://ecdc.europa.eu/en/publications-data/tularaemia-annual-epidemiological-report-2015

Tick-borne encephalitis - Annual Epidemiological Report for 2015

Date of publication: 30 January 2018

https://ecdc.europa.eu/en/publications-data/tick-borne-encephalitis-annual-epidemiological-report-2015

Congenital toxoplasmosis - Annual Epidemiological Report for 2015

Date of publication: 30 January 2018

https://ecdc.europa.eu/en/publications-data/congenital-toxoplasmosis-annual-epidemiological-report-2015

Dengue fever - Annual Epidemiological Report for 2015

Date of publication: 30 January 2018

https://ecdc.europa.eu/en/publications-data/dengue-fever-annual-epidemiological-report-2015

Ebola and Marburg fevers - Annual Epidemiological Report for 2015

Date of publication: 30 January 2018

https://ecdc.europa.eu/en/publications-data/ebola-and-marburg-fevers-annual-epidemiological-report-2015

Malaria - Annual Epidemiological Report for 2015

Date of publication: 30 January 2018

https://ecdc.europa.eu/en/publications-data/malaria-annual-epidemiological-report-2015

Shigatoxin/verocytotoxin-producing *Escherichia coli* (STEC/VTEC) infection - Annual Epidemiological Report for 2015

Date of publication: 30 January 2018

https://ecdc.europa.eu/en/publications-data/shigatoxinverocytotoxin-producing-escherichia-coli-stecvtec-infection-annual

Cryptosporidiosis - Annual Epidemiological Report for 2015

Date of publication: 26 January 2018

https://ecdc.europa.eu/en/publications-data/cryptosporidiosis-annual-epidemiological-report-2015

Chikungunya fever - Annual Epidemiological Report for 2015

Date of publication: 26 January 2018

https://ecdc.europa.eu/en/publications-data/chikungunya-fever-annual-epidemiological-report-2015

